# From raw data to meaningful information: a robust but flexible method to assess in vitro assay responses—lessons learned from a novel *Dicentrarchus labrax* estrogen screen test

**DOI:** 10.1007/s00204-026-04416-w

**Published:** 2026-05-05

**Authors:** Sylvain Slaby, Aurélie Duflot, Géraldine Maillet, Jérôme Couteau, Christophe Minier, Anne-Sophie Allonier-Fernandes, Patrícia I. S. Pinto, Thomas Knigge, Tiphaine Monsinjon

**Affiliations:** 1 INERIS, Normandie Univ, UMR-I 02 SEBIO, Université de Reims Champagne-Ardenne, Université Le Havre Normandie, Le Havre, F-76600 France; 2 TOXEM, Montivilliers, France; 3 Agence de l’eau Seine-Normandie, 12 rue de l’Industrie CS, Courbevoie Cedex, 80148 92416 France; 4 Laboratory of Comparative Endocrinology and Integrative Biology, Centre of Marine Sciences (CCMAR), Faro, Portugal

**Keywords:** Bioassays, Statistical analysis, Thresholds, Risk assessment

## Abstract

Statistical analysis of in vitro assay data is a critical step towards a good interpretation of biological responses. However, it is still frequently undermined due to inappropriate statistical procedures, misinterpretation, insufficient statistical power—often resulting from small sample sizes—or poorly defined methodologies, in case of standardized tests. In line with practices commonly adopted in clinical studies and more recently in biomonitoring research, the use of thresholds for interpreting results may improve the robustness of conclusions. This work presents the application of a methodology for defining thresholds using a normal distribution–based approach. As a case study, these thresholds were applied to analyze data obtained from the DLES test (*Dicentrarchus labrax* estrogen screen test), an in vitro screening tool designed to detect interactions between chemicals and nuclear estrogen receptors in *D. labrax*. The results were subsequently compared with methods derived from the OECD TG 455, as well as with non-parametric statistical analyses. By applying normal distribution–based thresholds, data analysis was simplified and the reliability of the DLES test results was increased, especially when compared with hypothesis tests. Also, this was especially true when studying non-model species, for which standard reference substances are rarely available. However, special attention should be paid to the size of the initial dataset used to define the thresholds. The methodology implemented here could provide insight for other in vitro assays. Overall, this article encourages the reflection on approaches to in vitro data analysis.

**Supplementary Information:**

The online version contains supplementary material available at 10.1007/s00204-026-04416-w.

## Introduction

Estrogen-like endocrine disruptor chemicals (EEDC) pose a serious threat to human and environmental health. Because of their varied physicochemical properties, they are widely distributed in all environmental compartments, especially within aquatic environments (Kabir et al. 2015; Monneret 2017; Thacharodi et al. 2023). Based on the Organization for Economic Co-operation and Development (OECD) test guidelines (TG), several bioassays were specifically designed for detecting compounds interacting with estrogen pathways (OECD, 2025). Most, if not almost all, are in vitro tests with human receptors (TG 455, 456, 457, and 493), while in vivo tests mainly employ rodent (TG 440) and fish (TG 229, 230, 250, and 252) model species. This focus neglects the complexity of signaling pathways across taxa, where the diversity in the type and number of nuclear and membrane receptors may be wide (Pinto et al. 2025; Serra et al. 2019, 2020; Slaby et al. 2024a; Sonavane et al. 2016; Zapater et al. 2024). For instance, while mammals possess two main nuclear receptor and one membrane, a set of three nuclear receptors (Esr1, Esr2a, and Esr2b) and two membrane receptors (GPER1 and GPER1-like) have been identified in most teleost fish (Lafont et al. 2016; Pinto et al. 2018; Zapater et al. 2024).

Although assessing risk for human health is a priority, the European Commission has stipulated the development of additional standardized screening tools that would enable a better characterization of EEDCs present in the environment (Grignard et al. 2022). Accordingly, various in vitro assays with aquatic vertebrate models have been developed using freshwater fish such as *Danio rerio*, *Oncorhynchus mykiss*, and *Oryzias latipes* (Slaby et al. 2024a). Yet, a few bioassays have also been developed for marine species, including *Micropogonias undulate*s (Kitano et al. 2006), *Paralichthys olivaceus* (Hawkins and Thomas 2004), *Sparus auratus* (Passos et al. 2009; Patrícia I. S. Pinto et al. 2006a, b), and *Dicentrarchus labrax* (Muriach et al. 2008; Pinto et al. 2019; Quesada-García et al. 2012; Zapater et al. 2019). Within this context and based on previous works, the DLES test was recently proposed as a reporter gene assay involving the three nuclear estrogen receptors identified in *D. labrax* (sbEsr1, sbEsr2a and sbEsr2b) (Slaby et al. 2024a).

In bioassay processing, data handling and, more particularly, statistical analyses constitute critical steps for the interpretation of the results. Yet, only few guidelines for standardized in vitro tests prescribe detailed procedures for handling and interpreting data (Hothorn 2014). Even if statistical significance tests are recommended and applied, they may be misused or fail to reflect a true biological meaning (Greenland et al. 2016; Hothorn 2014). Moreover, the “all or nothing” application of p-value thresholds (statistically significant or not) with excessive confidence in this parameter should be critically assessed and—perhaps—even abandoned altogether, as claimed by more than 800 experts from statistics, clinical and medical researches, biology, and psychology (Amrhein et al. 2019). It appears that data processing, and especially statistical analysis, are the “odd one out” in the standardization of screening bioassays. Standardized methodologies for analyzing in vitro bioassay data are lacking or, leastwise, are not sufficiently designated by clear guidance, which calls for a harmonization of good practices.

Threshold-based methodologies to analyze data have long been used in the field of human health. As an example, clinical hematological analyses are often linked to reference values. Kluxen and Hothorn (2020) raised a number of questions that need to be dealt with before translating such methods to other fields. These authors raised concern about the way reference values are established. Notably, they addressed the choice of historical control data versus concurrent controls and the selection of confidence, tolerance, or prediction intervals. For instance, the size of any historical dataset is required to be sufficiently large in order to reduce variance. Furthermore, the increase in response variability under contaminated conditions must also be considered. In developing a robust approach for defining limits of detection in bioassays, Holstein et al. (2015) stressed that accounting for variance under test conditions reduces the risk of both Type I and Type II errors, i.e., false positives and false negatives.

Accurate result interpretation from in vitro assays involves the use of analyzing tools that combine robustness and ease of use. This is even more important when tests are conducted following routine execution such as in vitro bioassays implying users with no particular statistical expertise. In this context, the use of thresholds appears to be a well-adapted approach, as they are clear-cut and, by consequence, univocal. For bioassays designed to assess alterations of the estrogen pathway, the OECD TG 455 guideline (i.e., transactivation in vitro assays to detect estrogen receptor agonists and antagonists) already relies on comparing results obtained under test conditions with reference values derived from the concentration range of positive controls, dedicated to assessing signal induction on the one hand and signal inhibition on the other hand. However, the guideline does not provide a procedure for detecting an increased response beyond that normally elicited by the natural ligand, i.e., response amplification. Moreover, the TG 455 methodology is based on interactions with human estrogen nuclear receptors, for which the literature provides extensive knowledge and for which well-characterized inhibitors are available.

A solution to the problem of determining a biologically meaningful response value could be provided by the normal distribution-based threshold. Such statistically determined thresholds have been employed by Leprêtre et al. (2022) for environmental biomonitoring in order to analyze biomarker responses in in situ caged organisms. The calculation of these thresholds relied on the work of Besse et al. (2013), who defined a normal distribution-based threshold in order to assess the bioaccumulation of contaminants in *Gammarus fossarum*. In brief, the calculation of this type of threshold relies on the assumption that biological data should—in general—follow a normal distribution. This normal distribution corresponds to the natural variation observed in a response or biological status. It is commonly referred to as ‘background noise’ in in vitro assays. Values that deviate significantly from this distribution are interpreted as being beyond natural variability and, hence, can be attributed to stress-induced effects. Such thresholds were then successfully applied on large scale environmental biomonitoring along the freshwater-estuarine-coastal continuum of the Seine-Normandie Basin (Slaby et al. 2024b).

Here we propose that normal distribution-based thresholds can be adopted for in vitro screening assays in order to decide whether a signal is indeed meaningful, i.e., corresponds to an induction or inhibition of estrogenic signaling pathways (Besse et al. 2013). To this end, we applied this threshold determination to results obtained from an in vitro test, the DLES test – *Dicentrarchus labrax* Estrogen Screening (Slaby et al. 2024a). The methodology enabled the determination of thresholds for assessing activation of the estrogen pathway (induction threshold) as well as thresholds for detecting inhibition or enhancement of the E2-mediated activation of this pathway (inhibition and amplification thresholds, respectively). The results obtained by using these thresholds were then compared with thresholds obtained with the logistic response-based threshold definition of the OECD TG-455 guideline. In addition, a common statistical approach (Kruskal-Wallis test followed by Conover-Iman post-hoc comparisons) was included in this comparison. The outcomes of this comparison provide a robust and reliable procedure that may be used to analyze data from in vitro screening bioassays, whether standardized or still under development.

## Materials and methods

### Reagents and substances

Gibco^tm^ Dulbecco’s Modified Eagle Medium (DMEM, with high glucose, sodium pyruvate, GlutaMAX^tm^ and phenol red), Gibco™ DMEM Nutrient Mixture F-12 (DMEM/F-12, no phenol red), Gibco™ penicillin-streptomycin (10000 U.mL^− 1^), Gibco™ Opti-MEM™ I Reduced Serum Medium (no phenol red), Invitrogen™ Lipofectamine™ 3000 Transfection Reagent, Thermo Scientific™ 17-β estradiol (E2, purity: 98%) were purchased from Fisher Scientific (Waltham, USA). Fetal bovine serum (FBS, non-USA origin), charcoal stripped FBS (South America origin) and dimethyl sulfoxide (DMSO) were obtained from Dutscher (Bernolsheim, France). DI-dithiothreitol 98% (HPLC) and adenosine 5-triphosphate disodium salt were both purchased from Sigma-Aldrich (Saint-Quentin-Fallavier, France) and d-luciferin potassium salt from Revvity (Bussy St Martin, France).

### The DLES test

The DLES test is an in vitro bioassay based on a reporter gene system that evaluates the estrogenic activity of compounds alone or in mixtures. It is based on the activation of one of the three nuclear estrogen receptors of *D. labrax* (sbEsr1, sbEsr2a, and sbEsr2b) and the expression of a reporter gene encoding luciferase under the control of an estrogen response element (ERE). The procedure used in this study was slightly modified compared to Slaby et al. (2024) in order to shorten its duration and is detailed in Supplementary Information (Appendix A.1.). Briefly, HEK 293 cells (ATCC CRL-1573) were transfected using the Lipofectamine™ 3000 Transfection Reagent kit following the manufacturer’s recommendations. Regarding the exposure, compounds or environmental contaminant extracts were tested alone, diluted at different range concentration or in mixture with E2 (10^− 8^ M). All exposure solutions contained 0.1% DMSO. Per plate, negative controls (NC, 0.1% DMSO) and a range of the positive control E2 (10^− 11^, 10^− 10^, 10^− 9^, 10^− 8^, 10^− 7^, 10^− 6^ M) were prepared in order to obtain quality control for the experiment. The 10^− 8^ M of E2 condition represented the positive control (PC) because it was determined as the lowest E2 concentration for which induction of luciferase activity has been observed for sbEsr1, sbEsr2a, and sbEsr2b (Slaby et al. 2024a). Each exposure was performed in triplicate (24 h, 37 °C, 5% CO_2_). After the exposure, the firefly luciferase assay reagent, developed by Siebring-Van Olst et al. (2013), was used in order to assess the luciferase activity. The luminescence intensity was measured using the luminometer function of a multi-well plate reader (2000 ms, 24 °C, Tecan Infinite^®^ M200, Männedorf, Switzerland).

### Data processing and statistical analysis

#### Normalization of raw data

For each 96-well plate, the different concentrations of the tested solution were analyzed in triplicate. The luminescence data were processed as follows: the average of the NC was considered as background noise and subtracted from all measurements. The corrected values were then normalized relative to the PC. All data analyses described below were performed using RStudio (Build 576).

#### Normal distribution-based threshold definition

Normal distribution-based thresholds were generated using the procedure described byLeprêtre et al. (2022) by adapting the calculation fromBesse et al. (2013) with minor modifications. In this study, the method was used to establish three distinct thresholds: (i) the induction threshold, which indicates a significant increase in luciferase activity; (ii) the inhibition threshold, which indicates a significant reduction in the E2-mediated induction of luciferase activity, and (iii) the amplification threshold, which indicates a significant enhancement of the E2-mediated induction of luciferase activity. Data used to determine the thresholds were obtained from environmental sample extracts collected in the Normandy region (Orne river, 49°09’08.0"N 0°22’57.0"W) or from chemicals exposures, such as bisphenol A (BPA).

**Fig. 1:**
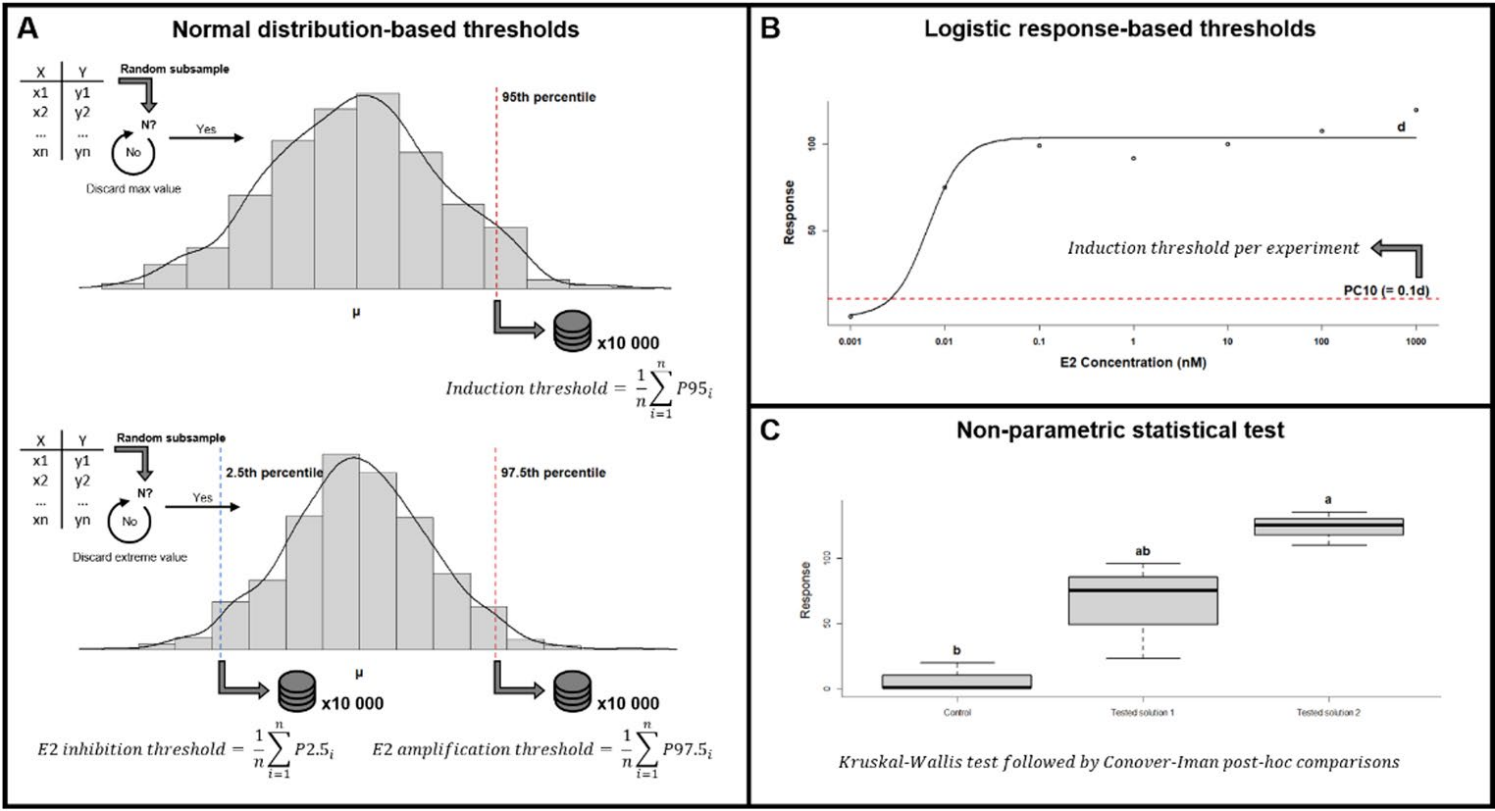
Schematic representation of the different analytical approaches used in this study.** A** Determination of normal distribution-based thresholds for induction, inhibition and amplification of the response.** B** Determination of the logistic response-based threshold; PC10 corresponds to 10% of the maximal response d (i.e., 0.1d).** C** Application of classical hypothesis testing to identify statistically significant differences compared with control conditions

To define the induction threshold (Fig. 1A), all available data were compiled according to the type of receptor. After averaging the exposure triplicates of the luciferase measurements, the sample sizes were *n* = 1845 for sbEsr1, *n* = 1821 for sbEsr2a, and *n* = 1789 for sbEsr2b. As each set of samples was tested at various concentrations and several times, the means for each condition was determined providing a final dataset of *n* = 707 for sbEsr1, *n* = 699 for sbEsr2a, and *n* = 698 for sbEsr2b. Considering each receptor, 30 values of each individual receptor data set were randomly selected without replacement, and this subdataset was analyzed using the Shapiro-Wilk test to determine whether its distribution follows the normal distribution (α = 5%). If the test outputs did not deviate significantly from a normal distribution, the 95th percentile of the subdataset distribution was determined and compiled. Otherwise, if the condition of normal distribution was not met, the maximal value outside the normal distribution was removed successively until the null hypothesis of no significant difference from normal distribution could be accepted (Fig. 1A). Then, the entire process was reiterated 10 000 times for the normally distributed datasets and the mean ± standard error (SE) of all 95th percentiles was calculated. This mean constituted the induction threshold that enables to determine an estrogenic activity.

Concerning the inhibition and the amplification thresholds, only the means from data obtained with the condition combining E2 10^− 8^ M were considered. As described above, since each set of samples was assessed at multiple concentrations and repeatedly, the mean value for each condition was calculated, resulting in final datasets of *n* = 352 for sbEsr1, *n* = 351 for sbEsr2a, and *n* = 346 for sbEsr2b. Similarly, if the null hypothesis was rejected after applying the Shapiro-Wilk test, the most extreme absolute value of the subdataset was removed and the normal distribution was retested. Once the hypothesis of normal distribution was validated, the 2.5th and 97.5th percentiles were compiled. As proceeded earlier, this sequence was repeated 10 000 times and the mean ± SE of the sets of the 2.5th and 97.5th percentiles were calculated to define inhibition and amplification thresholds, respectively (Fig. 1A).

The rule of decision for concluding the presence or absence of estrogenic, anti-estrogenic, or amplified estrogenic activity required similar conclusions (i.e., above or below the threshold) from two experimental repetitions. Otherwise, the experiment was repeated another time.

#### Logistic response-based threshold definition

The logistic response-based thresholds were determined using a process similar to that described in OECD TG 455 guidelines (OECD, 2021; Fig. 1B). For each receptor and each experiment, results obtained following exposure to a range of E2 concentrations were used to establish a four-parameter logistic curve (4PL). Then, the maximum horizontal asymptote values (d) were extracted and divided by 10 to obtain the PC10 (10% positive control), the induction threshold used to define an estrogenic effect.

As discussed below in this article, for the estrogenic effects of the contaminant mixture extracted from an environmental sample mixed with E2 at 10⁻⁸ M, the OECD TG 455 guideline provides a method to determine anti-estrogenic activity, but it does not allow the assessment of a possible amplification of the E2-mediated induction. Therefore, the OECD TG 455 guideline recommends the use of a concentration range of the well-characterized human estrogen receptor inhibitor tamoxifen, with thresholds derived using a 4PL model to conclude on antagonistic effects. However, such a *modus operandi* requires a well characterized inhibitor molecule, which is rarely the case for non-model organisms. Hence, the adoption of the TG 455 guideline approach is compromised for antagonistic effects, i.e., receptor inhibition, in an in vitro bioassay with non-human receptors, such as the DLES test.

#### Non-parametric statistical test

Another simple method—universally known among scientists—would be to apply groupwise statistical tests, such as ANOVA or Kruskal-Wallis with appropriate *post-hoc* tests and p-value correction for multiple comparison (Fig. 1C). Our analytical approach involves performing a standard statistical test: the Kruskal-Wallis test followed by a *post-hoc* Conover-Iman test with Benjamini-Hochberg p-value adjustment method (α = 5%). It is widely used as an alternative to ANOVA when the assumptions normality and homoscedasticity cannot be reliably met, as in the present work. The Conover–Iman test was preferred to the Dunn test because it offers greater statistical power and better control of Type I error in *post-hoc* multiple comparisons when the number of groups exceeds five.

### Application to case studies

In order to evaluate and compare the advantages and limitations of the different above-described methods, they were applied to data collected from experiments using a known identified EEDC (BPA) and an environmental extract, both evaluated at various concentrations. In the first experiment, four concentrations of BPA, combined with and without the natural ligand E2 (10⁻⁸ M), were tested using the DLES assay at 10, 100, 1000 and 10,000 nM engulfing environmental realistic concentrations.

Subsequently, the above described data processing methods were also applied to the screening of a freshwater sample. It was collected (2 L) from the Seine River (49°20’18.9"N 1°05’48.6"E, Oissel, France) in January 2024. Contaminant extraction was performed using hydrophilic-lipophilic balance cartridges, which allows for capturing a broad spectrum of molecules, excluding only extremely polar and extremely non-polar compounds. Successive elutions were carried out with acetone and methanol. The eluates were then evaporated under vacuum. Extracted substances were dissolved in DMSO at a final concentration 2000 times higher than the environmental concentration (2000X). The extract was then tested at four concentrations: 0.01X, 0.1X, 1X, and 10X, both with and without E2 (10^−8^ M).

## Results and discussion

### Comparison of the normal distribution-based thresholds and the logistic response-based thresholds

**Fig. 2:**
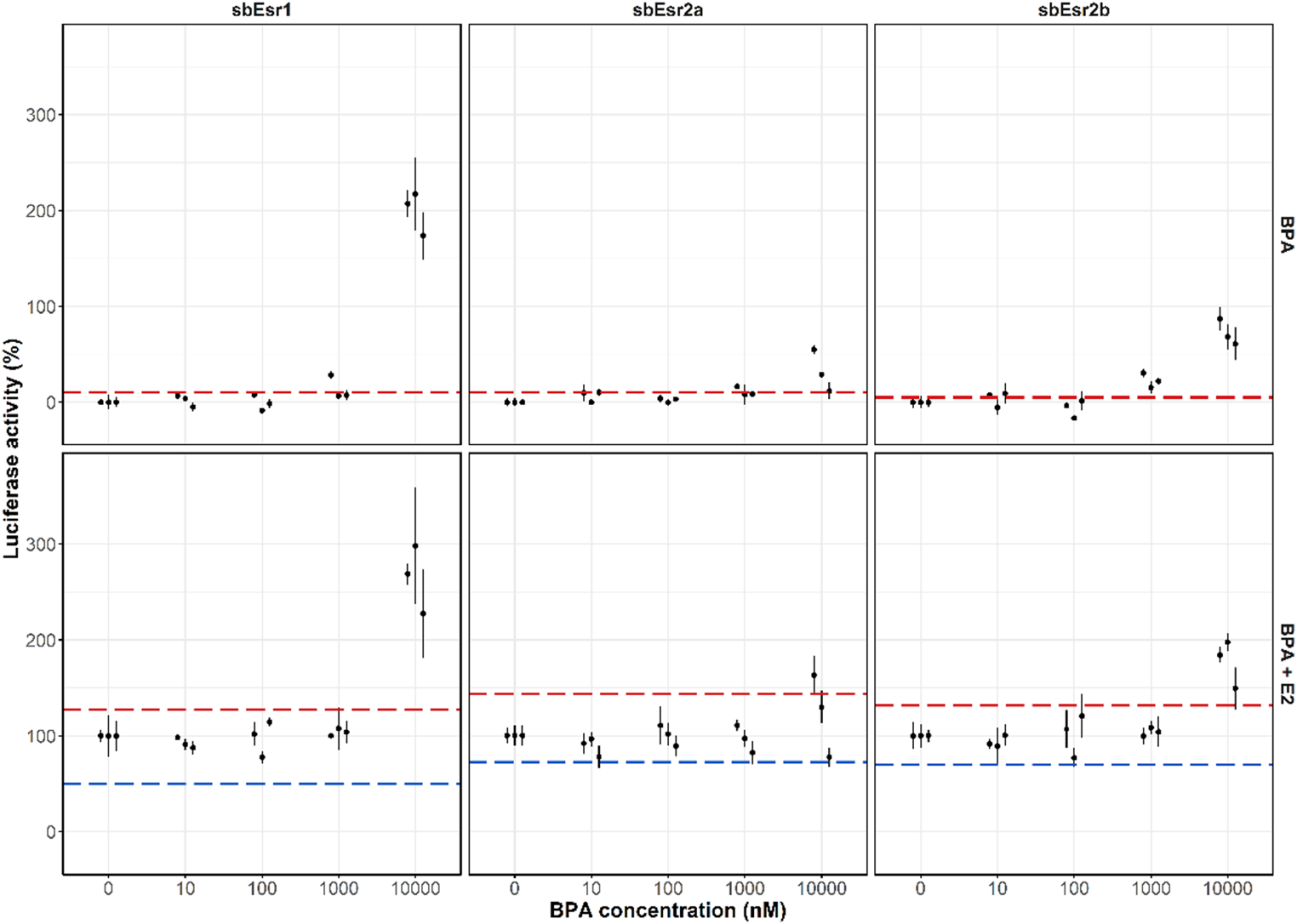
Luciferase activities measured using the DLES test with sbEsr1, sbEsr2a, and sbEsr2b following exposure to bisphenol A (BPA) and analyzed using the normal distribution-based thresholds. Each dot represents an individual experiment. Results are expressed as mean ± SD of experimental triplicates. Top panels: Exposure to BPA alone. The red line indicates the induction threshold. Positive luciferase induction is confirmed when at least two dots exceed this threshold. Bottom panels: Exposure to BPA in combination with 10 nM E2. Red and blue lines represent the E2 amplification and inhibition thresholds, respectively. Positive E2 amplification is confirmed when at least two dots exceed the red line, whereas positive E2-mediated inhibition is confirmed when at least two dots fall below the blue line

**Fig. 3:**
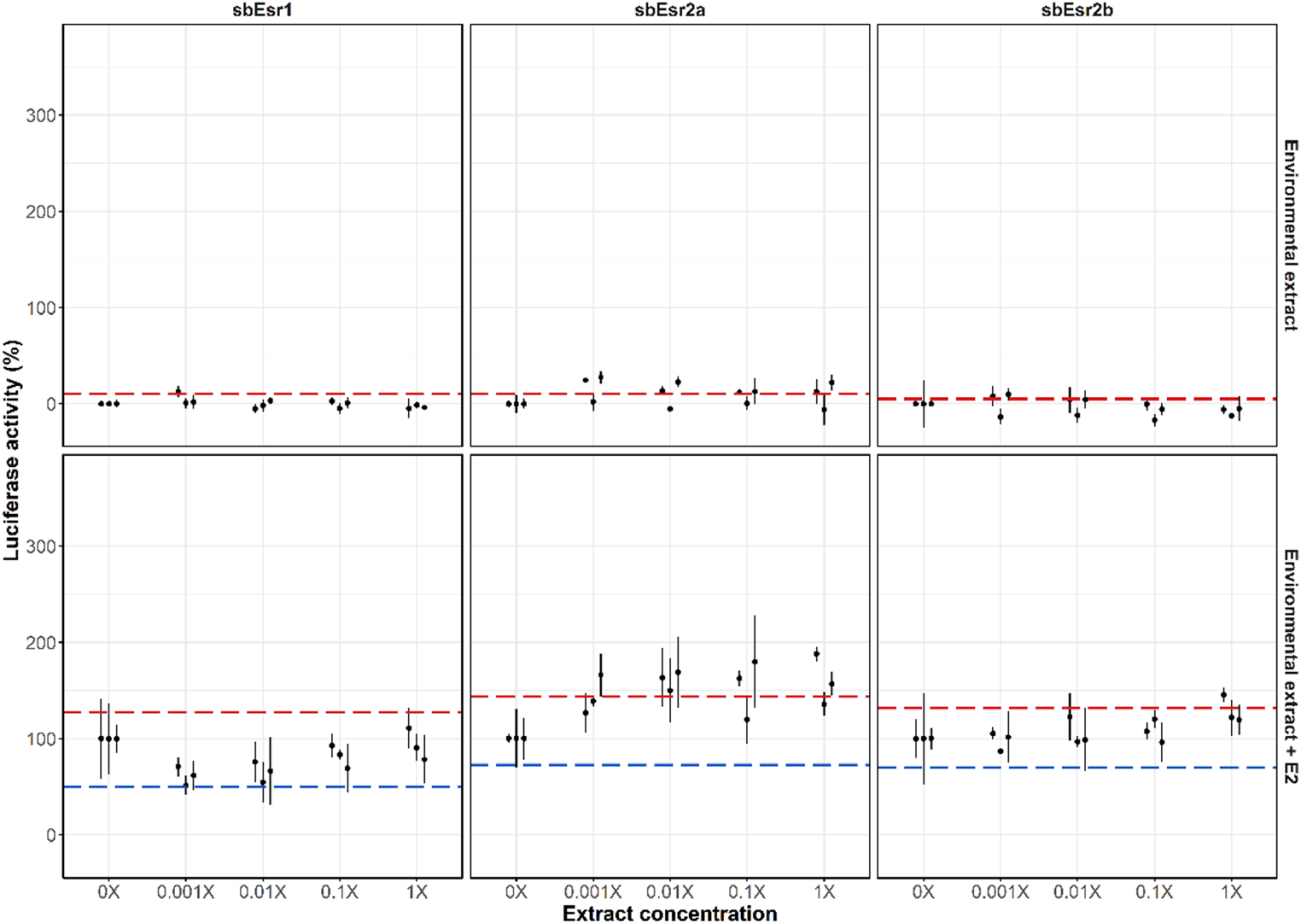
Luciferase activities measured using the DLES test with sbEsr1, sbEsr2a, and sbEsr2b following exposure to the environmental extract and analyzed using the normal distribution-based thresholds. Each dot represents an individual experiment. Results are expressed as mean ± SD of experimental triplicates. Top panels: Exposure to the environmental extract alone. The red line indicates the induction threshold. Positive luciferase induction is confirmed when at least two dots exceed this threshold. Bottom panels: Exposure to the environmental extract in combination with 10 nM E2. Red and blue lines represent the E2 amplification and inhibition thresholds, respectively. Positive E2 amplification is confirmed when at least two dots exceed the red line, whereas positive E2-mediated inhibition is confirmed when at least two dots fall below the blue line

**Fig. 4:**
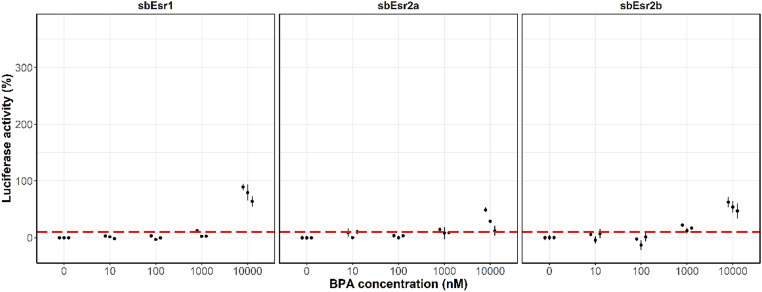
Luciferase activities measured using the DLES test with sbEsr1, sbEsr2a, and sbEsr2b following exposure to bisphenol A (BPA) and analyzed using the logistic response-based thresholds. Each dot represents an individual experiment. Results are expressed as mean ± SD of experimental triplicates. The red line indicates the induction threshold. Positive luciferase induction is confirmed when at least two dots exceed this threshold

**Fig. 5:**
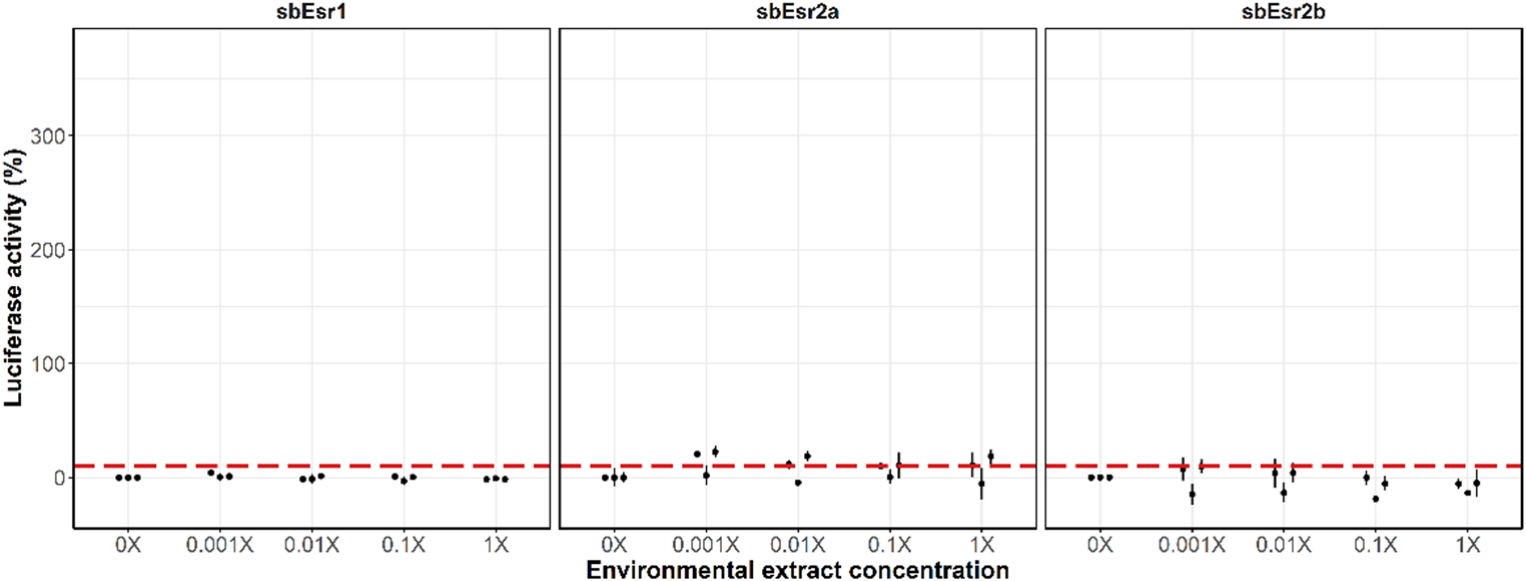
Luciferase activities measured using the DLES test with sbEsr1, sbEsr2a, and sbEsr2b following exposure to the environmental extract and analyzed using the logistic response-based thresholds. Each dot represents an individual experiment. Results are expressed as mean ± SD of experimental triplicates. The red line indicates the induction threshold. Positive luciferase induction is confirmed when at least two dots exceed this threshold

As depiected in Figs. 2 and 3, thresholds determined using the normal distribution-based threshold approach demonstrated that BPA induced an increase in estrogenic signaling *via* sbEsr1 (10000 nM), sbEsr2a (10000 nM) and sbEsr2b (10, 1000 and 10000 nM), while the environmental contaminant extract induced a response *via* both sbEsr2a (at all concentrations) and sbEsr2b (0.001X). Similar results were obtained with the logistic response-based thresholds after BPA exposures (Fig. 4). However, a slight difference can be noted for the environmental contaminant extract: no induction was detected with sbEsr2b (Fig. 5). Three main hypotheses could explain these outcomes. Firstly, the normal distribution-based thresholds are more sensitive for detecting exposure induction responses. Conversely, since the logistic response-based thresholds are primarily driven by the maximum value obtained within the E2 concentration range, any alteration in this concentration range could artificially raise or lower the threshold, potentially leading to false negative or false positive results, respectively. At last, the definition of specific thresholds for each receptor based on their response variabilities (normal distribution-based thresholds), considering their own specificities and sensitivities, could provide a more reliable means of discriminating estrogenic activities, compared to the somewhat arbitrary 10% maximal response threshold used in the logistic response-based thresholds.

For risk assessment, however, it may not be too problematic to observe discrepancies for the sbEsr2b response between the two methodologies. In fact, both classified the environmental location as impacted, since the presence and effects of estrogenic compounds were detected, no matter the concentration. Yet, such differences might become critical if the objective would be to characterize a known substance or mixture response, for which accurate quantification of effective concentrations is needed.

As mentioned above, no inhibition of the estrogenic signaling pathways was observed in any of the case studies. When the dataset is sufficiently large, the definition of specific thresholds, as proposed with the normal distribution-based thresholds, can replace the need for a well-defined antagonist response control, as these thresholds are based on the variation in the responses observed under conditions containing E2 (10 nM). In fact, the inhibition of estrogenic activity in bioassays that involve the human receptor can be assessed using well-known estrogen receptor antagonists (i.e., tamoxifen). Nevertheless, further studies are required to fully characterize the dose-response effects of any possible antagonist candidates and to evaluate their suitability as a reference compound for defining inhibition of estrogenic activity. The application of those reference compounds that are commonly applied in human studies needs be done with caution, as receptor responses can vary considerably between species. When moving to non-model organisms, taxa- or group-specific differences in estrogenic signaling may occur. This highlights the need for a careful, taxon-specific evaluation of the estrogen receptor agonists and antagonists commonly used in mammalian systems, in order to avoid erroneous assumptions and inappropriate extrapolations regarding the conservation of signaling mechanisms (Pinto et al. 2025). For instance, ICI 182,780, referred as a full antagonist in mammals, can act instead as a selective estrogen receptor modulator in other taxa, including environmentally relevant organisms such as teleost fish, where it may exert antagonist, agonist or amplification effects (Notch and Mayer 2011; Passos et al. 2009; Patrícia I.S. Pinto et al. 2006a, b; Slaby et al. 2024a). Another illustrative example concerns the human estrogen receptor antagonists methyl-piperidino-pyrazole and pyrazolo [1, 5-a] pyrimidine, which failed to inhibit the action of EE2 on the nuclear estrogen receptors of *D. rerio*, while co-exposure in assays resulted in an enhancement of EE2-mediated responses (Notch and Mayer 2011).

The normal distribution-based threshold approach concluded to an increase of the E2-induced estrogenic activity following exposures to 10,000 nM of BPA *via* sbEsr1 and sbEsr2b (Fig. 2), and also at 0.01X, 0.1X, and 1X of environmental extract *via* sbEsr2a (Fig. 3). In addition, endocrine disruption can be not only due to antagonists that interfere with the normal response to ligand–receptor binding, as previously mentioned, but also due to substances that amplify the response by acting directly on the receptor or on downstream signal transduction pathways. This mechanism is, however, not addressed by the available standardized assays, such as OECD TG 455, owning to the current lack of a well-characterized reference molecules capable of eliciting this specific effect on estrogen receptors.

### Comparison of the normal distribution-based thresholds and non-parametric statistical tests

To evaluate whether variations in a response or biological state is significantly different from a positive or negative control, statistical test procedures are required. The choice of an appropriate test depends on the nature of the data and on their variability. Parametric tests are preferred for their stronger statistical power, but non-parametric tests are frequently employed when the sample size (*n*) is low, as is the case in the examples presented in this study—a situation that is often found in bioassays. On the one hand, the limited *n* limits testing for normality and homoscedasticity. Reduced statistical power, however, tends to decrease the likelihood of detecting a truly significant difference.

The employment of statistical tests is often promoted to analyze in vitro test data. In fact, it is quite simple to measure the same endpoints in concurrent controls, because the medium in which the tested compound is diluted serves as negative control. However, several publications have emphasized that these tests can be easily misused and that p-values are often overrated (Amrhein et al. 2019; Greenland et al. 2016; Hothorn 2014; Thiese et al. 2015). Importantly, these methodologies rely on sufficient experimental replicates to draw robust conclusions, in accordance with result variability. However, achieving an adequate number of replicates can be challenging, even in high-throughput screening workflows.

**Fig. 6:**
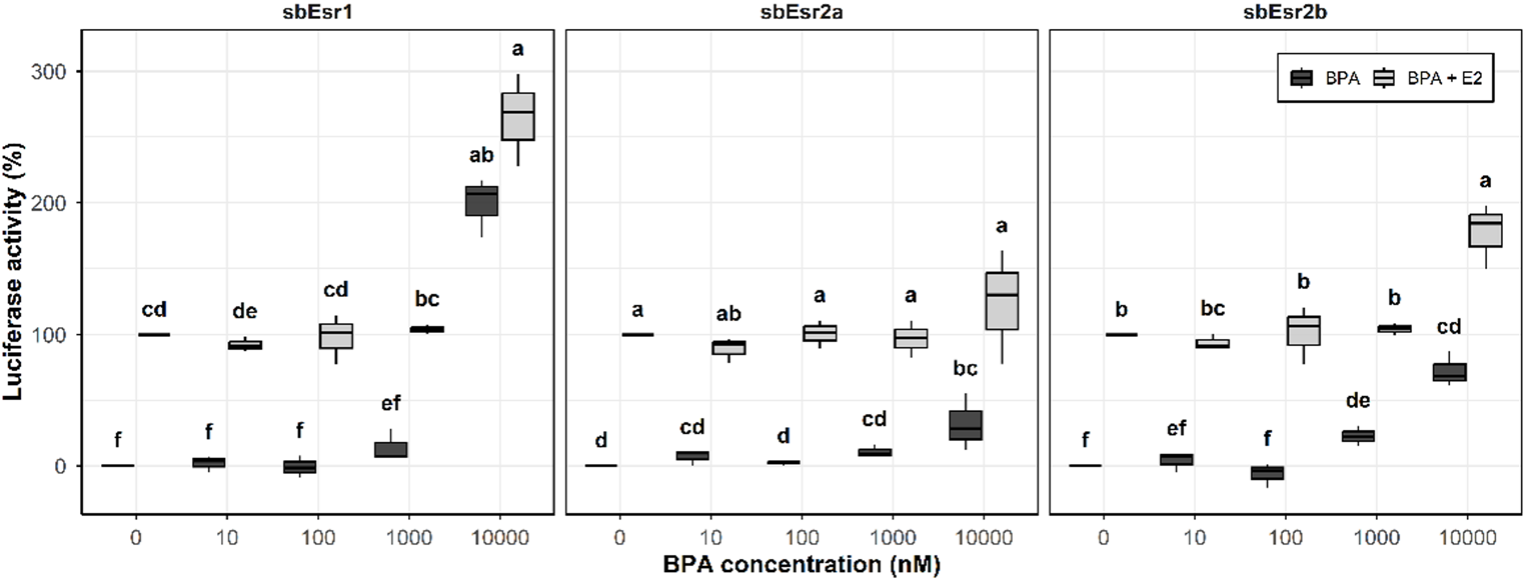
Luciferase activities measured using the DLES test with sbEsr1, sbEsr2a, and sbEsr2b following exposure to bisphenol A (BPA) and analyzed using the Kruskal-Wallis test followed by a post-hoc Conover-Iman test with Benjamini-Hochberg p-value adjustment method (α = 5%). Results are expressed as boxplot (central line: median value, box: the interquartile range, whiskers: minimum and maximum values). Different letters indicate significant differences

**Fig. 7:**
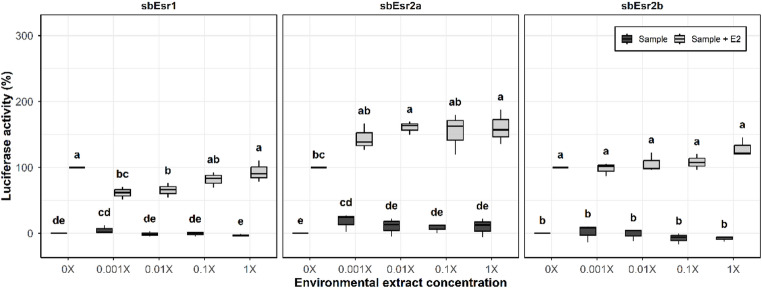
Luciferase activities measured using the DLES test with sbEsr1, sbEsr2a, and sbEsr2b following exposure to the environmental extract and analyzed using the Kruskal-Wallis test followed by a post-hoc Conover-Iman test with Benjamini-Hochberg p-value adjustment method (α  = 5%). Results are expressed as boxplot (central line: median value, box: the interquartile range, whiskers: minimum and maximum values). Different letters indicate significant differences

As shown in Fig. 6, in the absence of E2, the non-parametric tests conducted in this study only revealed a significant induction of the response at 10,000 nM BPA compared to the negative control for sbEsr1 (*p*-value = 1.79e-07), sbEsr2a (*p*-value=0.004), and sbEsr2b (*p*-value = 0.002), but also at 1,000 nM BPA compared to the negative control for sbEsr2b (*p*-value = 0.035). In the same way, they highlighted a significant induction at 0.001X of environmental extract with sbEsr2a (*p*-value = 0.036, Fig. 7). Under exposure conditions combining E2, an E2-induced response amplification at 10,000 nM BPA for sbEsr1 and sbEsr2b (*p*-value = 0.0003 and 0.007, respectively, Fig. 6), and at 0.01X and 1X of environmental extract with sbEsr2a were revealed (*p*-value = 0.008 and 0.0006, respectively, Fig. 7). These significant differences were also detected with the normal distribution-based threshold methodology. However, the non-parametric procedure failed to detect other relevant variations (Figs. 2 and 3). Applying the Benjamini-Hochberg correction to the p-values derived from the Conover–Iman tests resulted in a loss of statistical power, whereas alternative corrections would increase the risk of type I errors or decrease even more the statistical power. Nevertheless, the main reason for the lack of detection remains the low number of replicates (*n* = 3) associated to the response variability, a limitation frequently encountered in in vitro screening workflows.

Interestingly, the non-parametric tests also found a significant inhibition of the E2-induced response at 0.001X and 0.01X of the environmental extract with sbEsr1 (*p*-value = 7.3e-05 and 3.7e-06, respectively, Fig. 7). These effects were not shown by the normal distribution-based thresholds methodology (Fig. 3). Distinguishing true variations from natural biological variability remains challenging, especially for conventional statistical analyses which only take this variability into account with a very small data sample. In contrast, the normal distribution-based thresholds methodology eliminates these false-positive results by accounting for the natural variability of the response, including when the baseline activity is low.

### The definition of thresholds: validation and recommendations

The thresholds, obtained with Besse et al. (2013) and Leprêtre et al. (2022) methodology, were herein called normal distribution-based thresholds, defining an estrogenic effect with the contaminant extract. These were 10.16% for sbEsr1, 10.23% for sbEsr2a, and 5.02% for sbEsr2b. For the assessment of either inhibition and increase of the E2-induced response were 49.55 and 127.41% for sbEsr1, 72.59 and 143.66% for sbEsr2a, and 69.70 and 131.61% for sbEsr2b, respectively (Table 1).

The use of thresholds is recommended; however, their determination must be conducted appropriately. Alternative methods can be applied if deemed more appropriate for the data characteristics, such as in cases where the data do not follow a normal distribution in absence of pressures. For example, Besse et al. (2013) proposed also to fit data to the Baranyi model. It was applied because increasing contaminant concentrations in organisms sampled in situ can exhibit a distribution similar to a bacterial growth model. In this case, the *lag* parameter was used as the threshold.

An imperative for the application of the normal distribution-based threshold definition is a sufficiently large dataset in order to obtain a robust threshold (Leprêtre et al. 2022). Large datasets better reflect the intrinsic sensitivity of each receptor and reduced variability. Realistically, even though experimental conditions of bioassays are rigorously controlled, small variations in parameters such as room temperature, cell quality, cell culture medium quality, or pipetting precision cannot be fully avoided and may lead to small differences in the responses. Moreover, by integrating all data measured under both control and exposed conditions, the normal distribution-based threshold methodology accounts for potential changes in response variability induced by exposure during in vitro tests (Holstein et al. 2015).

**Table 1:** Normal distribution-based thresholds determined for each receptor and associated IC99.9

Receptors	Thresholds (Mean ± SE)	IC_99.9_
*Induction*		
sbEsr1	10.16 ± 0.07	[9.92, 10.39]
sbEsr2a	10.23 ± 0.04	[10.11, 10.35]
sbEsr2b	5.02 ± 0.04	[4.90, 5.14]
*E2-mediated inhibition*		
sbEsr1	49.55 ± 0.08	[49.28, 49.82]
sbEsr2a	72.59 ± 0.05	[72.42, 72.77]
sbEsr2b	69.70 ± 0.07	[69.46, 69.93]
*E2-mediated amplification*		
sbEsr1	127.41 ± 0.12	[127.01, 127.85]
sbEsr2a	143.66 ± 0.11	[143.28, 144.03]
sbEsr2b	131.61 ± 0.08	[131.35, 131.87]

SE: Standard error, IC_99.9_: 99.9% confidence interval

Before standardization, bioassays should be validated through rigorous procedures to characterize response variability and the intended scope of the test. This step offers the opportunity to accumulate sufficient data to set up thresholds that can subsequently be used for response analysis. As a key factor, the dataset size must be tailored to the response variability. In this work, we benefited from large datasets principally obtained from an environmental quality assessment study. However, the size should not depend solely on the number of available observations at the moment of analysis, but be guided by indicators of the reliability of the calculated mean. As shown in Table 1, the widths of the 99.9% confidence interval of the means are less than 1% for each threshold, indicating a good estimate.

As mentioned in Horowitz et al. (2010), who proposed a guideline to define, establish and verify reference intervals in the context of clinical assays, it is essential that threshold values would be transferable between laboratories. As such, certain requirements must be fulfilled: firstly, the analytical procedure should be consistent. Any modification to the protocol may require either a determination of new thresholds or, at least, a comparison of its influence on the results. As an example, in the context of a gene reporter assay, genes of interest (herein, sbEsr1, sbEsr2a, sbEsr2b, and the reporter gene) can be expressed in various cell lines. Such modifications and, notably, the differences in the intrinsic dataset variability that they may introduce, should be thoroughly studied and understood. Secondly, Horowitz et al. (2010) highlight the importance of the comparability of the test subject population. This condition, however, primarily applies to in vivo assays and is of lesser concern for in vitro bioassays.

## Conclusions

This article advocates a user-friendly method that is potentially widely applicable in order to extract robust data analysis and interpretation of in vitro screening bioassays. The method from Besse et al. (2013), modified by Leprêtre et al. (2022) allows for the determination of thresholds in a simple way that account for experimental variability. Moreover, it enables the assessment of effects relative to the positive control response (either inhibition or amplification), which is very valuable when non-model species are employed, or, as in this case, receptors of non-model species may differ even if only slightly from those of well-described model species. It is noteworthy that this approach depends on the dataset variability and size. It requires the experimental procedure to be repeated several times, as should be done anyway when proposing a new bioassay.

The use of clear and standardized response criteria allows for easier comparison of results and a more efficient evaluation of effects. Robust in vitro analysis and interpretation of the results constitute an essential basis for guiding investigations. This step is a crucial part of an Adverse Outcome Pathway framework, ultimately contributing to a relevant and reliable risk assessment.

## Supplementary Information

Below is the link to the electronic supplementary material.

Supplementary Material 1

## References

[CR1] Amrhein V, Greenland S, McShane B (2019) Scientists rise up against statistical significance. Nature 567:305–307. 10.1038/D41586-019-00857-9;SUBJMETA 30894741 10.1038/d41586-019-00857-9

[CR2] Besse J-P, Coquery M, Lopes C, Chaumot A, Budzinski H, Labadie P, Geffard O (2013) Caged Gammarus fossarum (Crustacea) as a robust tool for the characterization of bioavailable contamination levels in continental waters: Towards the determination of threshold values. Water Res 47:650–660. 10.1016/j.watres.2012.10.024 23182666 10.1016/j.watres.2012.10.024

[CR3] Greenland S, Senn SJ, Rothman KJ, Carlin JB, Poole C, Goodman SN, Altman DG (2016) Statistical tests, P values, confidence intervals, and power: a guide to misinterpretations. Eur J Epidemiol 31:337–350. 10.1007/S10654-016-0149-3/METRICS 27209009 10.1007/s10654-016-0149-3 PMC4877414

[CR4] Grignard E, de Jesus K, Hubert P (2022) Regulatory Testing for Endocrine Disruptors; Need for Validated Methods and Integrated Approaches. Front Toxicol 3:69. 10.3389/FTOX.2021.821736 10.3389/ftox.2021.821736 PMC8915824 35295107

[CR5] Hawkins MB, Thomas P (2004) The Unusual Binding Properties of the Third Distinct Teleost Estrogen Receptor Subtype ERβa Are Accompanied by Highly Conserved Amino Acid Changes in the Ligand Binding Domain. Endocrinology 145:2968–2977. 10.1210/en.2003-0806 15001543 10.1210/en.2003-0806

[CR6] Holstein CA, Griffin M, Hong J, Sampson PD (2015) Statistical Method for Determining and Comparing Limits of Detection of Bioassays. Anal Chem 87:9795–9801. 10.1021/acs.analchem.5b02082 26376354 10.1021/acs.analchem.5b02082

[CR7] Horowitz GL, Sousan A, Boyd JC, Ferruccio C, Uttam G, Horn P, Pesce A, Sine HE, Zakowski J (2010) Defining, establishing, and verifying reference intervals in the clinical laboratory: approved guideline, 3rd edn. Clinical and Laboratory Standards Institute, Wayne, Pennsylvania, USA

[CR8] Hothorn LA (2014) Statistical evaluation of toxicological bioassays – a review. Toxicol Res (Camb) 3:418–432. 10.1039/C4TX00047A

[CR9] Kabir ER, Rahman MS, Rahman I (2015) A review on endocrine disruptors and their possible impacts on human health. Environ Toxicol Pharmacol 40:241–258. 10.1016/j.etap.2015.06.009 26164742 10.1016/j.etap.2015.06.009

[CR10] Kitano T, Koyanagi T, Adachi R, Sakimura N, Takamune K, Abe S-I (2006) Assessment of estrogenic chemicals using an estrogen receptor α (ERα)- and ERβ-mediated reporter gene assay in fish. Mar Biol 149:49–55. 10.1007/s00227-005-0206-z

[CR11] Kluxen FM, Hothorn LA (2020) Alternatives to statistical decision trees in regulatory (eco-)toxicological bioassays. Arch Toxicol 94:1135–1149. 10.1007/S00204-020-02690-W/TABLES/2 32193567 10.1007/s00204-020-02690-w

[CR12] Lafont A-G, Rousseau K, Tomkiewicz J, Dufour S (2016) Three nuclear and two membrane estrogen receptors in basal teleosts, Anguilla sp.: Identification, evolutionary history and differential expression regulation. Gen Comp Endocrinol 235:177–191. 10.1016/j.ygcen.2015.11.021 26654744 10.1016/j.ygcen.2015.11.021

[CR13] Leprêtre M, Geffard A, Palos Ladeiro M, Dedourge-Geffard O, David E, Delahaut L, Bonnard I, Barjhoux I, Nicolaï M, Noury P, Espeyte A, Chaumot A, Degli-Esposti D, Geffard O, Lopes C (2022) Determination of biomarkers threshold values and illustration of their use for the diagnostic in large-scale freshwater biomonitoring surveys. Environ Sci Eur 34:115. 10.1186/s12302-022-00692-2

[CR14] Monneret C (2017) What is an endocrine disruptor? C R Biol 340:403–405. 10.1016/j.crvi.2017.07.004 29126512 10.1016/j.crvi.2017.07.004

[CR15] Muriach B, Cerdá-Reverter JM, Gómez A, Zanuy S, Carrillo M (2008) Molecular characterization and central distribution of the estradiol receptor alpha (ERα) in the sea bass (Dicentrarchus labrax). J Chem Neuroanat 35:33–48. 10.1016/j.jchemneu.2007.05.010 17629451 10.1016/j.jchemneu.2007.05.010

[CR16] Notch EG, Mayer GD (2011) Efficacy of pharmacological estrogen receptor antagonists in blocking activation of zebrafish estrogen receptors. Gen Comp Endocrinol 173:183–189. 10.1016/j.ygcen.2011.05.008 21641908 10.1016/j.ygcen.2011.05.008

[CR17] OECD (2021) Test No. 455: performance-based test guideline for stably transfected transactivation in vitro assays to detect estrogen receptor agonists and antagonists. OECD Guidelines for the Testing of Chemicals, Sect. 4, OECD Guidelines for the Testing of Chemicals, Sect. 4. 10.1787/9789264265295-en

[CR18] OECD (2025) Endocrine disrupters | OECD [WWW Document]. URL (Accessed 9.8.25) https://www.oecd.org/en/topics/sub-issues/testing-of-chemicals/endocrine-disrupters.html

[CR19] Passos ALS, Pinto PIS, Power DM, Canario AVM (2009) A yeast assay based on the gilthead sea bream (teleost fish) estrogen receptor β for monitoring estrogen mimics. Ecotoxicol Environ Saf 72:1529–1537. 10.1016/j.ecoenv.2009.02.004 19303142 10.1016/j.ecoenv.2009.02.004

[CR23] Pinto PatríciaIS, Passos AL, Rute SM, Power DM, Canário AVM (2006a) Characterization of estrogen receptor βb in sea bream (Sparus auratus): Phylogeny, ligand-binding, and comparative analysis of expression. Gen Comp Endocrinol 145:197–207. 10.1016/j.ygcen.2005.08.010 16213504 10.1016/j.ygcen.2005.08.010

[CR24] Pinto PatríciaIS, Singh PB, Condeça JB, Teodósio HR, Power DM, Canário AVM (2006b) ICI 182,780 has agonistic effects and synergizes with estradiol-17 beta in fish liver, but not in testis. Reprod Biol Endocrinol 4(14):67. 10.1186/1477-7827-4-67 10.1186/1477-7827-4-67 PMC1769500 17192186

[CR20] Pinto PIS, Andrade AR, Estêvão MD, Alvarado MV, Felip A, Power DM (2018) Duplicated membrane estrogen receptors in the European sea bass (Dicentrarchus labrax): Phylogeny, expression and regulation throughout the reproductive cycle. J Steroid Biochem Mol Biol 178:234–242. 10.1016/j.jsbmb.2017.12.019 29288793 10.1016/j.jsbmb.2017.12.019

[CR21] Pinto PIS, Andrade AR, Moreira C, Zapater C, Thorne MAS, Santos S, Estêvão MD, Gomez A, Canario AVM, Power DM (2019) Genistein and estradiol have common and specific impacts on the sea bass (Dicentrarchus labrax) skin-scale barrier. J Steroid Biochem Mol Biol 195:105448. 10.1016/j.jsbmb.2019.105448 31421232 10.1016/j.jsbmb.2019.105448

[CR22] Pinto PIS, Miglioli A, LaLone CA, Baumann L, Baynes A, Blanc-Legendre M, Cancio I, Cousin X, Dang ZC, Dumollard R, Ford AT, Green C, Iguchi T, Kearney P, Knigge T, Minier C, Monsinjon T, Monteiro MS, Sturve J, Watanabe H, Yamamoto H, Ankley G, Power DM, Katsiadaki I (2025) Prioritising research on endocrine disruption in the marine environment: a global perspective. Biol Rev. 10.1111/brv.70106 41290225 10.1111/brv.70106 PMC12965855

[CR25] Quesada-García A, Valdehita A, Fernández-Cruz ML, Leal E, Sánchez E, Martín-Belinchón M, Cerdá-Reverter JM, Navas JM (2012) Assessment of estrogenic and thyrogenic activities in fish feeds. Aquaculture 338–341:172–180. 10.1016/j.aquaculture.2012.02.010

[CR27] Serra H, Scholze M, Altenburger R, Busch W, Budzinski H, Brion F, Aït-Aïssa S (2019) Combined effects of environmental xeno-estrogens within multi-component mixtures: Comparison of in vitro human- and zebrafish-based estrogenicity bioassays. Chemosphere 227:334–344. 10.1016/j.chemosphere.2019.04.060 30999174 10.1016/j.chemosphere.2019.04.060

[CR26] Serra H, Brion F, Chardon C, Budzinski H, Schulze T, Brack W, Aït-Aïssa S (2020) Estrogenic activity of surface waters using zebrafish- and human-based in vitro assays: The Danube as a case-study. Environ Toxicol Pharmacol 78:103401. 10.1016/j.etap.2020.103401 32417722 10.1016/j.etap.2020.103401

[CR28] Siebring-Van Olst E, Vermeulen C, De Menezes RX, Howell M, Smit EF, Van Beusechem VW (2013) Affordable luciferase reporter assay for cell-based high-throughput screening. J Biomol Screen 18:453–461. 10.1177/1087057112465184 23112084 10.1177/1087057112465184

[CR29] Slaby S, Duflot A, Zapater C, Gómez A, Couteau J, Maillet G, Knigge T, Pinto PIS, Monsinjon T (2024a) The Dicentrarchus labrax estrogen screen test: A relevant tool to screen estrogen-like endocrine disrupting chemicals in the aquatic environment. Chemosphere 362:142601. 10.1016/j.chemosphere.2024.142601 38880263 10.1016/j.chemosphere.2024.142601

[CR30] Slaby S, Geffard A, Fisson C, Bonnevalle-Normand M, Allonier-Fernandes A-S, Amara R, Bado-Nilles A, Bonnard I, Bonnard M, Burlion-Giorgi M, Cant A, Catteau A, Chaumot A, Costil K, Coulaud R, Delahaut L, Diop M, Duflot A, Geffard O, Jestin E, Le Foll F, Le Guernic A, Lopes C, Palos-Ladeiro M, Peignot Q, Poret A, Serpentini A, Tremolet G, Turiès C, Xuereb B (2024b) Advancing environmental monitoring across the water continuum combining biomarkers in multiple sentinel species: A case study in the Seine-Normandie Basin. J Environ Manage 358:120784. 10.1016/j.jenvman.2024.120784 10.1016/j.jenvman.2024.120784 38603847

[CR31] Sonavane M, Creusot N, Maillot-Maréchal E, Péry A, Brion F, Aїt-Aïssa S (2016) Zebrafish-based reporter gene assays reveal different estrogenic activities in river waters compared to a conventional human-derived assay. Sci Total Environ 550:934–939. 10.1016/j.scitotenv.2016.01.187 26851879 10.1016/j.scitotenv.2016.01.187

[CR32] Thacharodi A, Hassan S, Hegde TA, Thacharodi DD, Brindhadevi K, Pugazhendhi A (2023) Water a major source of endocrine-disrupting chemicals: An overview on the occurrence, implications on human health and bioremediation strategies. Environ Res 231:116097. 10.1016/J.ENVRES.2023.116097 37182827 10.1016/j.envres.2023.116097

[CR33] Thiese MS, Arnold ZC, Walker SD (2015) The misuse and abuse of statistics in biomedical research. Biochem Med (Zagreb) 25:5–11. 10.11613/BM.2015.001/FULLARTICLE 25672462 10.11613/BM.2015.001 PMC4401313

[CR34] Zapater C, Molés G, Muñoz I, Pinto PIS, Canario AVM, Gómez A (2019) Differential involvement of the three nuclear estrogen receptors during oogenesis in European sea bass (Dicentrarchus labrax)†. Biol Reprod 100:757–772. 10.1093/biolre/ioy227 30371737 10.1093/biolre/ioy227

[CR35] Zapater C, Moreira C, Knigge T, Monsinjon T, Gómez A, Pinto PIS (2024) Evolutionary history and functional characterization of duplicated G protein-coupled estrogen receptors in European sea bass. J Steroid Biochem Mol Biol 236:106423. 10.1016/j.jsbmb.2023.106423 37939740 10.1016/j.jsbmb.2023.106423

