# Supplementary material for: From raw data to meaningful information: a robust but flexible method to assess in vitro assay responses—lessons learned from a novel Dicentrarchus labrax estrogen screen test
Source: Arch Toxicol. 2026 May 5;100(8):3583–94. doi: 10.1007/s00204-026-04416-w (PMC13379407; doi:10.1007/s00204-026-04416-w)
Supplement: Supplementary file 1 — Supplementary Material 1 [file 204_2026_4416_MOESM1_ESM.docx]

**Archives of Toxicology**

**From raw data to meaningful information: a robust but flexible method to assess *in vitro* assay responses – lessons learned from a novel *Dicentrarchus labrax* estrogen screen test**

**Supplementary information**

Sylvain Slaby^a*^, Aurélie Duflot^a^, Géraldine Maillet^b^, Jérôme Couteau^b^, Christophe Minier^a^, Anne-Sophie Allonier-Fernandes^c^, Patrícia I.S. Pinto^d^, Thomas Knigge^a^, Tiphaine Monsinjon^a*^

^a^ Université Le Havre Normandie, Université de Reims Champagne-Ardenne, INERIS, Normandie Univ, UMR-I 02 SEBIO, F-76600 Le Havre, France

^b^ TOXEM, Montivilliers, France

^c^ Agence de l'eau Seine-Normandie, 12 rue de l'Industrie CS 80148 92416 Courbevoie Cedex, France

^d^ Laboratory of Comparative Endocrinology and Integrative Biology, Centre of Marine Sciences (CCMAR), Faro, Portugal

* Corresponding authors.

E-mail addresses: sylvain.slaby@univ-lehavre.fr (S. Slaby), tiphaine.monsinjon@univ-lehavre.fr (T. Monsinjon)

Normandie Univ, UNILEHAVRE, FR CNRS 3730 SCALE, UMR-I 02 Environmental Stress and Aquatic Biomonitoring (SEBIO), Le Havre, France

**Summary:**

| **Appendix A.1.** | Detailed methodology of the DLES test. | p. 2 |
| --- | --- | --- |

**Appendix A.1.** Detailed methodology of the DLES test.

HEK 293 cells (ATCC CRL-1573) were cultured at 37 °C and 5% CO_2_ in DMEM supplemented with 10% FBS and 1% penicillin-streptomycin until 80-100% confluence. These cells were then resuspended in DMEM/F12 medium (10% charcoal stripped FBS + 1% penicillin-streptomycin) and seeded in 24-well plates (Starlab, Orsay, France) at a concentration of 125000 cells per well (200 µL). Lipotransfection was performed using the Lipofectamine™ 3000 Transfection Reagent kit following the manufacturer's recommendations (0.75 µg DNA per well, 24 h, 37 °C, 5% CO₂). Twenty-four hours after transfection, the medium in each well was replaced with 600 µL of DMEM/F12 solution (10% charcoal stripped FBS + 1% penicillin-streptomycin). The cells were resuspended and, after homogenization, 100 µL was transferred to white 96‑well polystyrene microplates (Revvity, Bussy St Martin, France), and incubated for further 24 hours (37 °C, 5% CO₂).

Regarding the exposure, compounds or environmental contaminant extracts were tested alone, diluted at different range concentration or in mixture with E2 (10^-8^ M). All exposure solutions contained 0.1% DMSO. Per plate, negative controls (NC, 0.1% DMSO) and a range of the positive control E2 (10^‑11^, 10^-10^, 10^-9^, 10^-8^, 10^-7^, 10^-6^ M) were prepared in order to obtain quality control for the experiment. The 10^-8^ M of E2 condition represented the positive control because it was determined as the lowest E2 concentration for which induction of luciferase activity is observed for sbEsr1, sbEsr2a, and sbEsr2b (Slaby et al., 2024). Each exposure was performed in triplicate (24 h, 37 °C, 5% CO_2_).

After the exposure, firefly luciferase assay reagent (FLAR, 10 mM MgCO_2_, 200 mM tricine, 50 mM MgSO_4_, 10 mM ethylenediaminetetraacetic acid, 500 mM dithiothreitol, 10 mM luciferin, 10 mM adenosine triphosphate), developed by Siebring-Van Olst et al. (2013), was used in order to assess the luciferase activity. After equilibrating the temperatures of the media containing the exposed cells and the FLAR solution to 24 °C, 100 µL of this preparation was added to each well. After 5 min of incubation in the dark at 24 °C, the luminescence intensity was measured using the luminometer function of a multi-well plate reader (2000 ms, 24 °C, Tecan Infinite® M200, Männedorf, Switzerland).

**References:**

Siebring-Van Olst, E., Vermeulen, C., De Menezes, R. X., Howell, M., Smit, E. F., & Van Beusechem, V. W. (2013). Affordable luciferase reporter assay for cell-based high-throughput screening. *Journal of Biomolecular Screening*, *18*(4), 453–461. https://doi.org/10.1177/1087057112465184

Slaby, S., Duflot, A., Zapater, C., Gómez, A., Couteau, J., Maillet, G., Knigge, T., Pinto, P. I. S., & Monsinjon, T. (2024). The Dicentrarchus labrax estrogen screen test: A relevant tool to screen estrogen-like endocrine disrupting chemicals in the aquatic environment. *Chemosphere*, *362*, 142601. https://doi.org/10.1016/j.chemosphere.2024.142601
